# Influences on antidepressant prescribing trends in the UK: 1995–2011

**DOI:** 10.1007/s00127-016-1306-4

**Published:** 2016-11-24

**Authors:** Becky Mars, Jon Heron, David Kessler, Neil M. Davies, Richard M. Martin, Kyla H. Thomas, David Gunnell

**Affiliations:** 1 Centre for Academic Mental Health, School of Social and Community Medicine, University of Bristol, Oakfield House, Bristol, BS8 2BN UK; 2 MRC Integrative Epidemiology Unit, University of Bristol, Bristol, UK

**Keywords:** Antidepressants, General practice, Prescribing, Trends, Primary care

## Abstract

**Purpose:**

The number of antidepressants prescribed in the UK has been increasing over the last 25 years; however, the reasons for this are not clear. This study examined trends in antidepressant prescribing in the UK between 1995 and 2011 according to age, sex, and drug class, and investigated reasons for the increase in prescribing over this period.

**Methods:**

This is a retrospective analysis of antidepressant prescribing data from the Clinical Practice Research Datalink: a large, anonymised, primary care database in the UK. The dataset used in this study included 138 practices, at which a total of 1,524,201 eligible patients were registered across the 17-year period. The proportion of patients who received at least one antidepressant prescription and the number of patients who started a course of antidepressants were calculated for each year of the study. We used person years (PY) at risk as the denominator. The duration of treatment for those starting antidepressants was also examined.

**Results:**

23% of patients were prescribed an antidepressant on at least one occasion over the 17-year study period. Antidepressant prescriptions rose from 61.9 per 1000 PY in 1995 to 129.9 per 1000 PY in 2011. This was largely driven by an increase in prescribing of selective serotonin reuptake inhibitors and ‘other’ antidepressants. In contrast, incidence rates of those starting antidepressants remained relatively stable (1995: 21.3 per 1000 PY; 2011: 17.9 per 1000 PY). The duration of treatment increased with later starting years, with an increasing proportion of long-term use, and decrease in short-term use.

**Conclusion:**

The increase in antidepressant prescribing over the study period appears to be driven by an increase in long-term use of these medications.

**Electronic supplementary material:**

The online version of this article (doi:10.1007/s00127-016-1306-4) contains supplementary material, which is available to authorized users.

## Introduction

In the UK, antidepressant (AD) prescribing has increased substantiality over the past two decades, leading to concerns that they are being overprescribed. Similar increases have been reported in other European countries, the USA, Canada, and Australia [1–10]. There are a number of potential explanations for this rise, including improved recognition of depression, availability of new AD drugs, changes in patient/GP attitudes, and a broadening of the range of indications treated with ADs.

One particularly important question is whether the rise in AD prescribing can be attributed to more people starting on AD treatment. Previous studies that have investigated this issue have produced conflicting results, with some studies finding an increase in the number of people who have started taking ADs, and other studies finding that rates have remained stable, or even decreased [2, 4, 6, 8, 10–12]. There is also increasing evidence to suggest that the rise in AD prescribing is driven by an increase in long-term use [2, 4, 6–8, 12].

Existing studies investigating AD trends in the UK have often been restricted to specific regions [4, 13], spanned short-time periods, or have limited their analysis to patients with a diagnosis of depression [11, 12]. ADs are prescribed for a wide range of indications, and research suggests that a substantial proportion of patients prescribed them do not have a diagnosis of depression [14, 15]. To gain a more complete understanding of AD trends, it is, therefore, necessary to broaden analysis beyond patients with a depression diagnosis. Moreover, as GPs increasingly classify depression using symptom codes as opposed to diagnostic codes [16–18], restricting analysis to patients with a diagnosis may miss cases.

This paper examines trends in AD prescribing (regardless of indication) between 1995 and 2011 using data from the Clinical Practice Research Datalink (CPRD): a large, anonymised, primary care database in the UK. Our objectives were to:

- Examine trends in incidence (the number of patients starting on ADs) and period prevalence (patients starting ADs plus existing AD users) over the study period, and investigate whether there are differences according to age, gender, and drug class.
- Examine trends in the duration of treatment amongst patients starting ADs.
- Explore the potential influence of external events that overlapped with our study period, including (1) the 2008 recession; (2) the 2006 quality outcomes framework (QOF); (3) the 2003 Medicines and Healthcare products Regulatory Agency (MHRA) advice against the use of selective serotonin reuptake inhibitors (SSRIs) other than fluoxetine in under 18s and (4) the introduction of the ‘Improving Access to Psychological Therapies’ (IAPT) initiative in 2006.

## Methods

### Data source

The Clinical Practice Research Datalink (CPRD) is one of the largest primary care databases in the world and contains anonymised electronic records from over 4 million active patients, representing 6.9% of the UK population (http://www.cprd.com). The CPRD contains information on diagnosis, symptoms, prescriptions, referrals and test results, as well as demographic and administrative information. These data are routinely entered by GPs and their staff onto their computer systems. Participating general practices use a computerised system called Vision, which has built-in software to extract and anonymise data from practice computers. The patient population captured in the database is broadly representative of the overall UK population in terms of age, sex and geographical distribution [19, 20].

### Study population

We examined trends in AD prescriptions issued in Primary Care between 1995 and 2011. We included all patients aged 14 and over who had been registered with a CPRD practice for at least 3 years; therefore, data were extracted from 1st January 1992. Analyses were restricted to ‘acceptable’ patient records from practices that met the CPRD quality criteria and contributed data for the entire study period. The dataset used in this study included 138 practices, at which a total of 1,524,201 eligible patients were registered across the 17-year period.

Patients under 14 years were excluded as AD prescribing is rare in younger children. A minimum period of 3 years registration was chosen to improve the identification of patients who started ADs (referred to as incident cases) Incident AD users were defined as those with no previous AD prescription during the study period and all incident cases had a minimum AD-free period of 3 years. For example, a patient prescribed an AD in July 2000 would have to be registered since at least July 1997 to be included in the year 2000 stats. If they had no prior prescriptions during the study period, they would be classified as an incident case for that year.

### Analysis

We identified all AD prescriptions (drugs included in “Comparison with existing literature” of the British National Formulary) [21] prescribed to patients between 1st January 1992 and 31st December 2011. These prescriptions were classified into three categories, based on their proposed method of action (Electronic Supplementary Material 1): selective serotonin reuptake inhibitors (SSRIs), tricyclic antidepressants (TCAs), and ‘other’ antidepressants, largely consisting of mirtazapine and venlafaxine (78%).

For each calendar year of the study, we calculated the number of patients who received at least one AD prescription, and the number of patients who started ADs. We used person years at risk (PY) as the denominator; for example, a patient who was registered for 3 months of the year and then left the practice (e.g. died, or transferred to a non CPRD-contributing practice) would contribute 0.25 years to the denominator for that year. Trends in AD prescribing were also examined separately by drug class and stratified according to age and sex.

Join point regression analysis [22] was used to estimate the years (with 95% CI) in which changes in trends occurred (software version 4.2. available from http://surveillance.cancer.gov/joinpoint/). The analysis involves fitting a series of joined straight lines, and selecting the point(s) at which the rate of increase/decrease changes significantly (join points). An annual percentage change (APC) is calculated for each of the identified trends, based on the slope of the line segment between join points.

Sensitivity analyses were conducted (1) excluding patients prescribed low doses (<75 mg) of amitriptyline, as this is commonly prescribed for indications other than depression, particularly pain [3, 5] and (2) amongst the subgroup of patients with a diagnosis of depression during the study period (see Electronic Supplementary Material 2 for a list of read codes [23] used to indicate a depression diagnosis).

To examine changes in long-term prescribing over time, we calculated the duration of incident treatment episodes. The intended duration of treatment was estimated from the dosing instructions and the quantity prescribed. Where no dosage instructions were provided, the median for the product type was used. Consecutive prescriptions were considered to be part of the same treatment episode if the gap between the expected end of one prescription and the start of another was less than 4 months. This was based on guidance from the ACNP task force [24] who suggest recovery is ascribed after at least 4 months following the onset of remission. Prescription duration was divided into the following six categories: ≤30, 31–60, 61–180, 181–365, 366–730, 731 days+. Consecutive prescriptions were not required to be the same product type. We use the phrase ‘long-term use’ to refer to prescriptions with a duration greater than 1 year.

## Results

There were 1,280,995 antidepressant prescribing events amongst 350,398 patients (23% of the total sample). The majority of the AD prescriptions were SSRIs (51%), with TCAs accounting for 40% and other ADs 9%. The number of patients who started ADs over the study period was 241,903.

### Prevalence of antidepressant usage

**Fig. 1:**
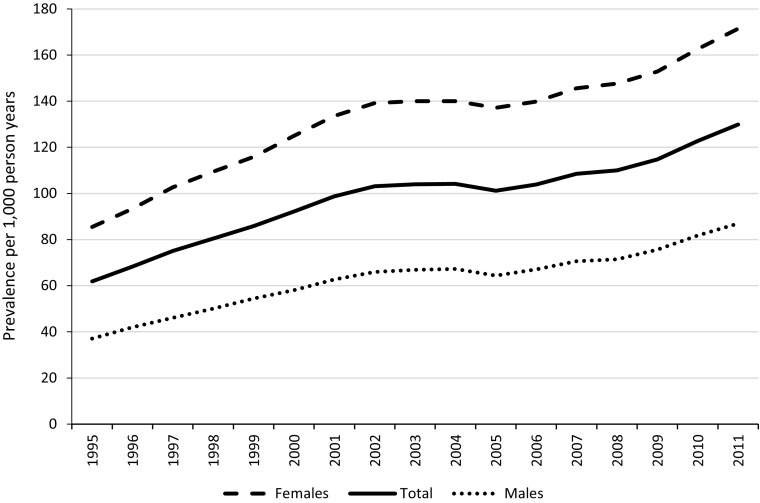
Prevalence of antidepressant prescriptions per 1000 person years

Figure 1 illustrates the period prevalence of antidepressant use per 1000 PY for each year of the study. AD prescriptions increased by more than 100%, rising from 61.9 per 1000 PY in 1995 to 129.9 per 1000 PY in 2011. In the join point analysis, the best fitting model included the following four join points (Supplementary Figure 1): 1997 (95% CI 1997–2000) when there was a reduction in the rate of increase in AD prescribing, 2002 (95% CI 2000–2003) after which AD prescribing levelled off, 2005 (95% CI 2004–2006) when prescribing rose again, and 2008 (95% CI 2007–2009) when the rate of prescribing accelerated. A similar pattern was observed for males and females, although AD prescribing was about two times greater in females.

**Fig. 2:**
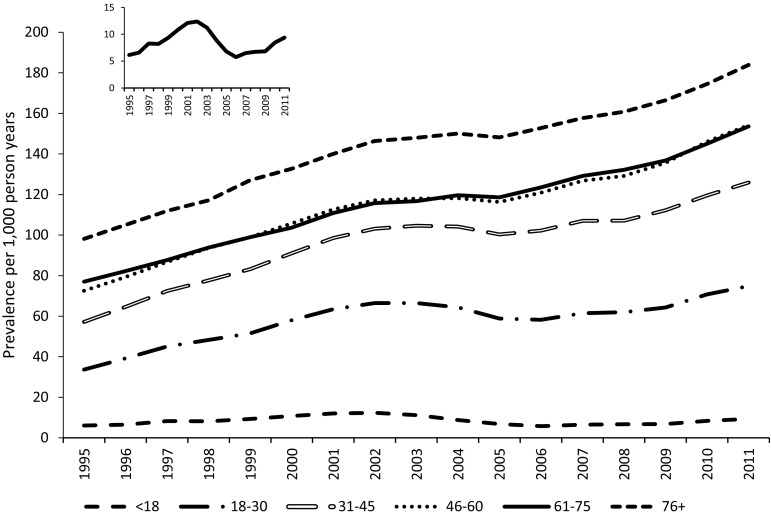
Prevalence of antidepressant prescriptions per 1000 person years, according to age strata

There was a progressive increase in the level of AD prescribing with increasing age (Fig. 2). The overall pattern of change between 1995 and 2011 was similar across the age strata, with the exception of the youngest age category (<18’s). For this group, we found a substantial drop in prevalence between 2002 and 2006, following which there was a steady increase (see inset in Fig. 2). There was also a notable drop in prevalence between 2002 and 2006 in the 18–30-year age group (from 66.5 to 58.3 per 1000 PY).

**Fig. 3:**
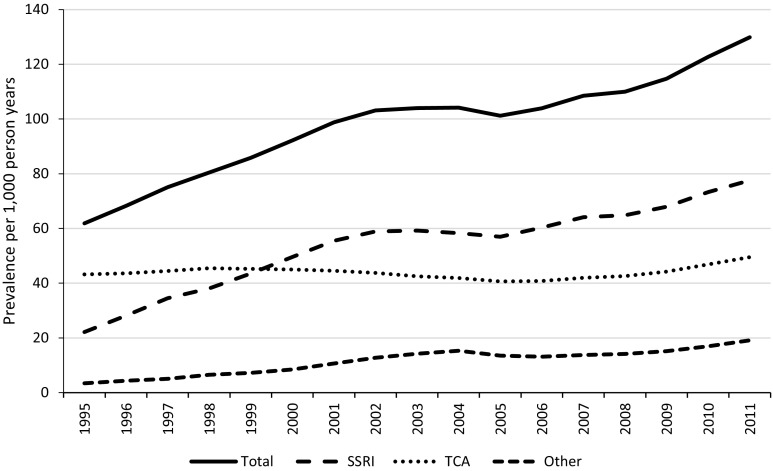
Prevalence of antidepressant prescriptions per 1000 person years, according to drug class

Figure 3 shows prevalence according to drug class. The increase in AD prescriptions was driven largely by a rise in prescriptions of SSRIs and ‘other’ ADs. In contrast, TCA prescriptions (56% amitriptyline) remained relatively stable.

### Incidence of antidepressant usage

**Fig. 4:**
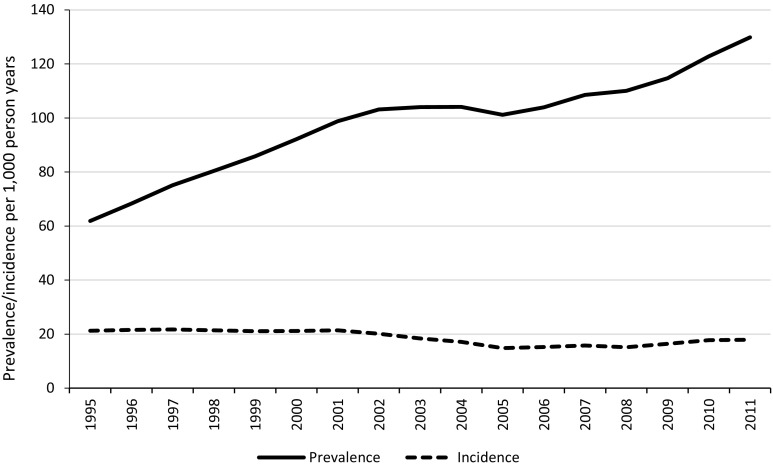
Prevalence and incidence of antidepressant prescriptions per 1000 person years

Figure 4 illustrates the number of patients starting ADs for the first time per 1000 PY for each year of the study. In contrast to the substantial rise in prevalence of AD use, the number of new cases has fallen slightly over time, from 21.3 per 1000 PY in 1995 to 17.9 per 1000 PY in 2011. The best fitting model included two join points; one in 2002 (95% CI 1999–2003), after which there was a substantial decline in incidence rates, and one in 2005 (95% CI 2004–2007) when incidence rates began to increase (Supplementary Figure 2). The overall pattern was similar for males and females (Supplementary Figure 3). Incidence rates remained higher in females throughout the study; however, the difference became less pronounced over time (the F:M ratio decreased from 1.9:1 in 1995 to 1.2:1 in 2011. Chi-square test of trend: *P* = <0.001).

The pattern of prescribing was found to differ according to AD drug class (Supplementary Figure 4). Incident prescribing of SSRIs increased between 1995 and 2001, whereas TCAs declined over this period. Incidence rates for other AD prescriptions remained relatively stable throughout the study period.

### Duration of incident prescriptions

**Fig. 5:**
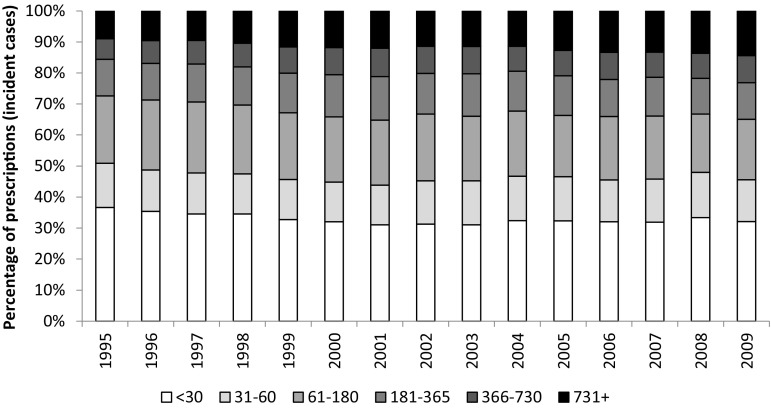
Changes in the proportion of patients with different treatment lengths between 1995 and 2009

We examined trends in the duration of treatment for patients starting ADs between 1995 and 2009. The median length of treatment increased over this period, from 44 to 56 days. Figure 5 shows the proportion of patients with different treatment lengths for each year of the study. We found an increasing proportion of long-term use with later starting years, and a corresponding decrease in short-term use.

Further information on treatment duration trends is provided in Supplementary Table 1, where results are presented separately according to drug class. For SSRIs and other AD prescriptions, the pattern of results was the same as for all ADs. For TCAs, there was an overall reduction in the length of treatment with later starting years; however, the proportion of patients prescribed TCAs for 2 years or more also increased slightly.

### Sensitivity analysis

#### Excluding patients prescribed low-dose amitriptyline

When excluding patients with low doses of amitriptyline (<75 mg) overall trends for prevalence and incidence (Supplementary Figures 5a and 5b) are similar to the main analysis. However, for TCAs, there was a substantial drop in prevalence and incidence rates over time, such that in later years, this drug class accounted for very few AD prescriptions.

#### Amongst the subgroup with depression

When restricting the sample to those with a diagnosis of depression (67% of patients prescribed an AD), a similar pattern of results was found to the main analysis. However, the drop in incidence of TCAs was more marked (Supplementary Figures 6a and 6b).

## Discussion

### Summary

Nearly a quarter of patients in the sample were prescribed an AD on at least one occasion during the study period. The prevalence of AD prescribing doubled between 1995 and 2011, although levels remained relatively stable between 2002 and 2005, when there was a notable reduction in prescribing to those under 30 years. The overall rise in prescribing was largely driven by an increase in SSRIs and other ADs. Our findings suggest the observed rise in prescribing is not due to an increased number of people starting meditation, but rather appears to be explained by an increase in the duration of treatment.

### Strengths and limitations

The study included data from a large anonymised database of Primary Care Patients, which enabled examination of AD trends according to drug class, and also by age and gender. Trends were also examined over a long period of time (17 years). We examined AD prescribing regardless of indication, which is important, given that ADs are prescribed for a range of indications other than depression; only 67% of the patients in our study had a depression-related Read code, and only 39% of patients who started on AD had one recorded in the year prior to their first prescription. Sensitivity analysis conducted in the subgroup of patients with depression and excluding those prescribed low doses of amitriptyline found similar results.

Findings must also be interpreted in light of several limitations. First, our analysis is based on prescriptions issued in Primary Care only, and we do not have information about the dispensing of medications or patient compliance. Second, although trends were examined over a long period of time, data were only available until 2011. Third, the results may not generalise to practices that do not contribute to the CPRD, or to other countries which have different healthcare systems. Fourth, when calculating duration of treatment, we chose a minimum period of 4 months between prescriptions to indicate the end of a treatment episode. However, we are unable to say whether patients actually achieved remission during the treatment period. Findings from sensitivity analysis using a minimum duration of 30 days and 6 months were similar (results available on request). Finally, incident AD users were defined as those with no previous AD prescription during the study period. This may have led to a bias of selecting proportionally more ‘real’ new starters and fewer re-starters in the later part of the follow-up period.

### Comparison with existing literature

The rise in prevalence of AD prescriptions found in this study is consistent with existing literature [1–10]. Studies regarding trends in incidence have been less consistent, with some studies reporting an increase in incident prescriptions over time [4, 6, 8], and others finding stable rates or a decrease [2, 10–12]. Our findings are also in line with previous reports that the rise in AD prescribing is due to an increase in the proportion of patients receiving long-term treatment [2, 4, 6–8, 12]. For example, a previous study using this database [11] found that the increase in AD prescriptions between 1993 and 2005 was explained by an increase in the proportion of patients receiving long-term prescriptions. Another UK study examining prescribing rates between 2003 and 2013 [12] found a reduction in AD prescribing for incident depression, and an increase in prescribing for recurrent depression. We extend these studies by examining trends over a longer time period, which overlaps with several important external events, and by not limiting our analysis to those with depression. Examining prescribing trends in the whole population is important, as a large proportion of patients prescribed ADs do not have a depression diagnosis. Moreover, GPs increasingly use symptom rather than diagnostic codes [16–18], which could result in cases being missed. There is also evidence to suggest that the introduction of QOF performance indicators for depression may have influenced prescribing [12]. This was found to be the case for both genders, and for both younger and older adults [12].

The prevalence and incidence of AD prescriptions was consistently higher amongst females than males. Although, as found in some previous studies [25, 26] there was a decrease in the ratio of female to male prescribing over time, indicating that AD prescribing has increased more in males. This could suggest there has been an increase in help-seeking behaviour in males, an increase in depression, or an increase in the number of males prescribed an AD for other indications.

The limited evidence on AD trends according to age suggests that AD prescriptions increase with increasing patient age [26–28]. We found an increase in prevalence of AD for all age groups, with the exception of those under 18 years [4, 5, 26]. Antidepressant prescribing in adolescents has received considerable attention following MHRA advice in 2003 against the initiation of SSRIs except fluoxetine in this age group. Consistent with our findings, studies from Europe, USA and Australia show that the regulatory warnings were associated with a reduction in the prescribing of ADs to children and adolescents [17, 29–33]. Our data also suggest that the warnings had a spill-over effect into other age groups [34, 35], with a join point indicating a change in trend in 2002. Several studies have found the reduction in AD prescribing following the regulatory warnings was not associated with a rise in adolescent suicides or non-fatal self-harm [31, 36, 37]. Rates began to rise following a second join point in 2005, which could suggest that concerns about a possible increased risk of suicidality have reduced.

We also explored whether trends in AD use were affected by a number of other events overlapping with the study period. These included the introduction of QOF performance indicators for depression in 2006, the 2008 recession, and improved access to cognitive behavioural therapy (CBT) through the IAPT initiative in 2006. Some studies have found an increase in AD prescribing or depression following the recession [18, 38]. For example, Kendrick et al. [18] found a rise in the prevalence of depression after 2008 in younger men, associated with increased unemployment. In this study, we found the best fitting model for trends in AD prevalence included a join point at 2008, following which the rate of prescribing accelerated. However, unlike Kendrick et al., our data suggest there was an increase in prevalence for both males and females, and for all age groups. Similar to previous research, we found no relationship between trends in AD prescribing and the introduction of IAPT services [38, 39], although it is possible that IAPT availability may have attenuated the recent rise in incident prescribing.

### Implications for research and practice

Guidelines for depression recommend that patients continue medication for at least 6 months after remission to reduce the risk of relapse [40]. Our findings of longer treatment periods for patients who began taking ADs in later years of the study suggest there is improved adherence to practice guidelines. While encouraging, it is important to note that the majority (65%) of patients who began AD treatment in 2009 discontinued treatment before the recommended time, with 32% of patients being prescribed ADs for 30 days or less. The increase in duration could also be attributed to the introduction of newer ADs, which may be better tolerated, or to changes in patient/GP attitudes regarding the treatment of mental illness. Alternatively, it could reflect failure by GPs to adequately follow-up patients and monitor treatment, with several studies finding that many patients on long-term AD treatment have not had a recent medication review [28, 41].

While long-term prescribing may be appropriate for some patients, currently little is known about the risks and benefits of taking AD medication long term. In the future, research, guidelines, and performance indicators should focus more on the appropriateness of long-term prescribing, and ensure regular review of patients who become established on long-term treatments.

## Electronic supplementary material

Below is the link to the electronic supplementary material.

Supplementary material 1 (DOCX 12 kb)

Supplementary material 2(DOCX 18 kb)

Supplementary material 3 (DOCX 99 kb)
