# Supplementary material for: Influences on antidepressant prescribing trends in the UK: 1995–2011
Source: Soc Psychiatry Psychiatr Epidemiol. 2016 Nov 24;52(2):193–200. doi: 10.1007/s00127-016-1306-4 (PMC5329088; doi:10.1007/s00127-016-1306-4)
Supplement: Supplementary file 1 — Supplementary material 1 (DOCX 12 kb) [file 127_2016_1306_MOESM1_ESM.docx]

**Appendix 1: Antidepressants according to drug class**

Selective Serotonin Reuptake Inhibitors (SSRIs)

citalopram hydrobromide, citalopram hydrochloride, escitalopram oxalate, fluoxetine hydrochloride, fluvoxamine maleate, paroxetine hydrochloride, sertraline hydrochloride

Tricyclic antidepressants (TCAs)

amitriptyline hydrochloride, amitriptyline hydrochloride/chlordiazepoxide, amitriptyline hydrochloride/perphenazine, amoxapine, clomipramine hydrochloride, desipramine hydrochloride, dosulepin hydrochloride, doxepin hydrochloride , fluphenazine hydrochloride/nortriptyline hydrochloride, imipramine hydrochloride, iprindole, lofepramine, maprotiline hydrochloride, mianserin hydrochloride, nortriptyline hydrochloride, protriptyline hydrochloride, trazodone hydrochloride, trimipramine maleate, viloxazine hydrochloride

Other antidepressants

agomelatine, duloxetine hydrochloride, flupentixol dihydrochloride, isocarboxazid, mirtazapine, moclobemide, nefazodone hydrochloride, phenelzine sulphate, reboxetine mesilate, tranylcypromine sulphate, trifluoperazine hydrochloride/tranylcypromine sulphate, tryptophan, venlafaxine hydrochloride
