# Supplementary material for: Influences on antidepressant prescribing trends in the UK: 1995–2011
Source: Soc Psychiatry Psychiatr Epidemiol. 2016 Nov 24;52(2):193–200. doi: 10.1007/s00127-016-1306-4 (PMC5329088; doi:10.1007/s00127-016-1306-4)
Supplement: Supplementary file 2 — Supplementary material 2(DOCX 18 kb) [file 127_2016_1306_MOESM2_ESM.docx]

**Appendix 2: Depression codes**

| **medcode** | **readcode** | **readterm** |
| --- | --- | --- |
| 324 | E2B..00 | Depressive disorder NEC |
| 12450 | 6896 | Depression screening using questions |
| 543 | Eu32z11 | [X]Depression NOS |
| 655 | E200300 | Anxiety with depression |
| 1996 | 1B17.00 | Depressed |
| 4824 | 1B17.11 | C/O - feeling depressed |
| 1131 | E204.00 | Neurotic depression reactive type |
| 4639 | Eu32.00 | [X]Depressive episode |
| 10015 | 1BT..00 | Depressed mood |
| 30405 | 9H92.00 | Depression interim review |
| 1908 | 2257 | O/E - depressed |
| 9796 | 1B1U.00 | Symptoms of depression |
| 2639 | E204.11 | Postnatal depression |
| 6932 | E113.11 | Endogenous depression - recurrent |
| 2970 | Eu32z00 | [X]Depressive episode, unspecified |
| 5987 | Eu32z14 | [X] Reactive depression NOS |
| 6950 | E112.13 | Endogenous depression first episode |
| 9211 | Eu32100 | [X]Moderate depressive episode |
| 595 | E112.14 | Endogenous depression |
| 5879 | E112.11 | Agitated depression |
| 1055 | E135.00 | Agitated depression |
| 4323 | E2B1.00 | Chronic depression |
| 10610 | E112.00 | Single major depressive episode |
| 11717 | Eu32000 | [X]Mild depressive episode |
| 6482 | E113700 | Recurrent depression |
| 19439 | 212S.00 | Depression resolved |
| 3292 | Eu33.00 | [X]Recurrent depressive disorder |
| 11913 | Eu41200 | [X]Mixed anxiety and depressive disorder |
| 10438 | 1B1U.11 | Depressive symptoms |
| 15099 | E113.00 | Recurrent major depressive episode |
| 3291 | Eu32z12 | [X]Depressive disorder NOS |
| 9667 | Eu32200 | [X]Severe depressive episode without psychotic symptoms |
| 6546 | E112.12 | Endogenous depression first episode |
| 9055 | Eu32.11 | [X]Single episode of depressive reaction |
| 10667 | Eu32400 | [X]Mild depression |
| 14709 | E113200 | Recurrent major depressive episodes, moderate |
| 2560 | E11..12 | Depressive psychoses |
| 16506 | E112100 | Single major depressive episode, mild |
| 7604 | Eu32.13 | [X]Single episode of reactive depression |
| 15220 | Eu34114 | [X]Persistant anxiety depression |
| 15155 | E112200 | Single major depressive episode, moderate |
| 1533 | E290.00 | Brief depressive reaction |
| 29520 | Eu33100 | [X]Recurrent depressive disorder, current episode moderate |
| 9183 | E11z200 | Masked depression |
| 7749 | Eu41211 | [X]Mild anxiety depression |
| 12099 | Eu32300 | [X]Severe depressive episode with psychotic symptoms |
| 8902 | Eu33.13 | [X]Recurrent episodes of reactive depression |
| 13307 | Eu53011 | [X]Postnatal depression NOS |
| 8851 | Eu33.11 | [X]Recurrent episodes of depressive reaction |
| 10455 | E211200 | Depressive personality disorder |
| 7011 | E112z00 | Single major depressive episode NOS |
| 16632 | E291.00 | Prolonged depressive reaction |
| 7737 | Eu34113 | [X]Neurotic depression |
| 25563 | E113z00 | Recurrent major depressive episode NOS |
| 17770 | E130.11 | Psychotic reactive depression |
| 6854 | Eu32y00 | [X]Other depressive episodes |
| 44300 | Eu33z00 | [X]Recurrent depressive disorder, unspecified |
| 29342 | E113100 | Recurrent major depressive episodes, mild |
| 29784 | Eu33000 | [X]Recurrent depressive disorder, current episode mild |
| 8584 | Eu34111 | [X]Depressive neurosis |
| 15219 | E112300 | Single major depressive episode, severe, without psychosis |
| 33469 | Eu33200 | [X]Recurr depress disorder cur epi severe without psyc sympt |
| 8478 | E130.00 | Reactive depressive psychosis |
| 22806 | Eu32212 | [X]Single episode major depression w'out psychotic symptoms |
| 25697 | E113300 | Recurrent major depressive episodes, severe, no psychosis |
| 11329 | Eu33211 | [X]Endogenous depression without psychotic symptoms |
| 34390 | E112000 | Single major depressive episode, unspecified |
| 47009 | Eu33300 | [X]Recurrent depress disorder cur epi severe with psyc symp |
| 55384 | E113600 | Recurrent major depressive episodes, in full remission |
| 19696 | Eu33.12 | [X]Recurrent episodes of psychogenic depression |
| 24112 | Eu32313 | [X]Single episode of psychotic depression |
| 24171 | E113400 | Recurrent major depressive episodes, severe, with psychosis |
| 43324 | E112500 | Single major depressive episode, partial or unspec remission |
| 18510 | Eu32.12 | [X]Single episode of psychogenic depression |
| 23731 | Eu33311 | [X]Endogenous depression with psychotic symptoms |
| 28248 | Eu32z13 | [X]Prolonged single episode of reactive depression |
| 16562 | Eu31300 | [X]Bipolar affect disorder cur epi mild or moderate depressn |
| 11252 | Eu33212 | [X]Major depression, recurrent without psychotic symptoms |
| 16861 | Eu33315 | [X]Recurrent severe episodes of psychotic depression |
| 32841 | 8HHq.00 | Referral for guided self-help for depression |
| 35671 | E113000 | Recurrent major depressive episodes, unspecified |
| 10720 | Eu32y11 | [X]Atypical depression |
| 32159 | E112400 | Single major depressive episode, severe, with psychosis |
| 32941 | Eu33313 | [X]Recurr severe episodes/major depression+psychotic symptom |
| 27491 | E11y200 | Atypical depressive disorder |
| 57409 | E112600 | Single major depressive episode, in full remission |
| 19054 | Eu3y111 | [X]Recurrent brief depressive episodes |
| 24117 | Eu32311 | [X]Single episode of major depression and psychotic symptoms |
| 28863 | Eu32314 | [X]Single episode of reactive depressive psychosis |
| 47731 | Eu33y00 | [X]Other recurrent depressive disorders |
| 36246 | E290z00 | Brief depressive reaction NOS |
| 41989 | Eu32211 | [X]Single episode agitated depressn w'out psychotic symptoms |
| 98252 | Eu32600 | [X]Major depression, moderately severe |
| 46244 | E02y300 | Drug-induced depressive state |
| 32845 | Eu92000 | [X]Depressive conduct disorder |
| 37764 | Eu33316 | [X]Recurrent severe episodes/reactive depressive psychosis |
| 98346 | Eu32500 | [X]Major depression, mild |
| 15923 | E115000 | Bipolar affective disorder, currently depressed, unspecified |
| 98414 | Eu32700 | [X]Major depression, severe without psychotic symptoms |
| 31757 | Eu33314 | [X]Recurr severe episodes/psychogenic depressive psychosis |
| 36616 | Eu33z11 | [X]Monopolar depression NOS |
| 56609 | Eu32y12 | [X]Single episode of masked depression NOS |
| 52678 | Eu32312 | [X]Single episode of psychogenic depressive psychosis |
| 59386 | Eu32213 | [X]Single episode vital depression w'out psychotic symptoms |
| 98417 | Eu32800 | [X]Major depression, severe with psychotic symptoms |
| 73991 | Eu33214 | [X]Vital depression, recurrent without psychotic symptoms |
| 46244 | E02y300 | Drug-induced depressive state |
| 32841 | 8HHq.00 | Referral for guided self-help for depression |
| 1531 | Eu31.11 | [X]Manic-depressive illness |
| 7953 | Eu34100 | [X]Dysthymia |
| 11596 | E11y000 | Unspecified manic-depressive psychoses |
| 12831 | E115.11 | Manic-depressive - now depressed |
| 4677 | E115.00 | Bipolar affective disorder, currently depressed |
| 33751 | Eu31z00 | [X]Bipolar affective disorder, unspecified |
| 56273 | E113500 | Recurrent major depressive episodes,partial/unspec remission |
| 60178 | E11y.00 | Other and unspecified manic-depressive psychoses |
| 44674 | E002.00 | Senile dementia with depressive or paranoid features |
| 23963 | ZV11111 | [V]Personal history of manic-depressive psychosis |
| 27759 | Eu02z16 | [X] Senile dementia, depressed or paranoid type |
| 57409 | E112600 | Single major depressive episode, in full remission |
| 29451 | Eu33213 | [X]Manic-depress psychosis,depressd,no psychotic symptoms |
| 30688 | Eu3y011 | [X]Mixed affective episode |
| 22080 | ZV11112 | [V]Personal history of manic-depressive psychosis |
| 28677 | Eu33312 | [X]Manic-depress psychosis,depressed type+psychotic symptoms |
| 23713 | Eu31400 | [X]Bipol aff disord, curr epis sev depress, no psychot symp |
| 44693 | Eu31600 | [X]Bipolar affective disorder, current episode mixed |
| 4732 | Eu31500 | [X]Bipolar affect dis cur epi severe depres with psyc symp |
| 35734 | E115100 | Bipolar affective disorder, currently depressed, mild |
| 20785 | Eu20400 | [X]Post-schizophrenic depression |
| 37296 | E115z00 | Bipolar affective disorder, currently depressed, NOS |
| 53840 | Eu31y00 | [X]Other bipolar affective disorders |
| 27890 | E115200 | Bipolar affective disorder, currently depressed, moderate |
| 41089 | E002z00 | Senile dementia with depressive or paranoid features NOS |
| 73924 | Eu31y11 | [X]Bipolar II disorder |
| 35607 | E115300 | Bipolar affect disord, now depressed, severe, no psychosis |
| 63701 | E115400 | Bipolar affect disord, now depressed, severe with psychosis |
| 57465 | E115600 | Bipolar affective disorder, now depressed, in full remission |
| 72026 | E115500 | Bipolar affect disord, now depressed, part/unspec remission |

*Read codes are a coded thesaurus of clinical terms used in [General Practice](https://en.wikipedia.org/wiki/General_Practice) in the United Kingdom. They provide the standard vocabulary by which clinicians can record patient findings and procedures in IT systems across primary and secondary care*
