# Supplementary material for: Influences on antidepressant prescribing trends in the UK: 1995–2011
Source: Soc Psychiatry Psychiatr Epidemiol. 2016 Nov 24;52(2):193–200. doi: 10.1007/s00127-016-1306-4 (PMC5329088; doi:10.1007/s00127-016-1306-4)
Supplement: Supplementary file 3 — Supplementary material 3 (DOCX 99 kb) [file 127_2016_1306_MOESM3_ESM.docx]

**Supplementary material**

**Title:** Influences on antidepressant prescribing trends in the UK: 1995-2011

**Journal:** Social Psychiatry and Psychiatric Epidemiology

**Authors:** Becky Mars*, Jon Heron, David Kessler, Neil M Davies, Richard M Martin, Kyla H Thomas, David Gunnell

* School of Social and Community Medicine, University of Bristol

Email: becky.mars@bristol.ac.uk

**Index of figures**

**Supplementary figure 1**: Best fitting join-point model of prevalence of antidepressant prescriptions per 1,000 person years

**Supplementary figure 2**: Best fitting join-point model of incidence of antidepressant prescriptions per 1,000 person years

**Supplementary figure 3**: Incidence of antidepressant prescriptions per 1,000 person years, according to gender

**Supplementary figure 4**: Incidence of antidepressant prescriptions per 1,000 person years, according to drug class

**Supplementary figure 5a**: Prevalence of antidepressant prescriptions per 1,000 person years, according to drug class, excluding those prescribed low doses of amitriptyline

**Supplementary figure 5b**: Incidence of antidepressant prescriptions per 1,000 person years, according to drug class, excluding those prescribed low doses of amitriptyline

**Supplementary figure 6a**: Prevalence of antidepressant prescriptions per 1,000 person years amongst those with a diagnosis of depression

**Supplementary figure 6b**: Incidence of antidepressant prescriptions per 1,000 person years, amongst those with a diagnosis of depression

Supplementary Table 1: changes in the proportion of patients with different treatment lengths between 1995 and 2009

| **Number of days** | **1995** | **2009** | **Absolute**  **percentage change** | **95% CI** | **P value *** | **Relative percentage change** |
| --- | --- | --- | --- | --- | --- | --- |
| **All AD** |  |  |  |  | < 0.001 |  |
| <30 | 36.63% | 32.09% | -4.54% | - 5.67% to -3.42% |  | -12.4% |
| 31-60 | 14.27% | 13.51% | -0.76% | -1.58% to 0.06% |  | -5.33% |
| 61-180 | 21.69% | 19.45% | \| -2.24% \|  \| \| --- \| --- \| | -3.19% to -1.2% |  | -10.33% |
| 181-365 | 11.79% | 11.79% | 0.00% | -0.76% to 0.76% |  | 0.00% |
| 366-730 | 6.65% | 8.74% | 20.9% | 1.45% to 2.72% |  | 31.43% |
| 731+ | 8.97% | 14.42% | 5.45% | 4.69% to 6.21% |  | 60.76% |
|  |  |  |  |  |  |  |
| **SSRIs** |  |  |  |  | < 0.001 |  |
| <30 | 35.57% | 26.20% | -9.37% | -11.04% to -7.70% |  | -26.34% |
| 31-60 | 10.44% | 8.44% | -2.00% | -3.06% to -0.94% |  | -19.16% |
| 61-180 | 23.51% | 20.49% | -3.02% | -4.52% to -1.52% |  | -12.85% |
| 181-365 | 13.25% | 15.19% | 1.94% | 0.69% to 3.19% |  | 8.25% |
| 366-730 | 7.79% | 11.93% | 4.14% | 3.09% to 5.19% |  | 14.64% |
| 731+ | 9.43% | 17.75% | 8.32% | 7.12% to 9.52% |  | 53.15% |
|  |  |  |  |  |  |  |
| **TCAs** |  |  |  |  | < 0.001 |  |
| <30 | 36.57% | 39.65% | 3.08% | 1.44% to 4.71% |  | 8.42% |
| 31-60 | 16.56% | 20.59% | 4.03% | 2.71% to 5.36% |  | 24.34% |
| 61-180 | 20.74% | 17.81% | -2.93 | -4.25% to -1.61% |  | -14.13% |
| 181-365 | 11.15% | 7.47% | -3.68 | -4.64% to -2.72% |  | -33.00% |
| 366-730 | 6.09% | 4.53% | -1.56 | -2.30% to -0.82% |  | -25.62% |
| 731+ | 8.89% | 9.94% | 1.05% | 0.06% to 2.04% |  | 11.81% |
|  |  |  |  |  |  |  |
| **Other AD** |  |  |  |  | < 0.001 |  |
| <30 | 46.76% | 32.20% | -14.56 | -20.26% to -8.86% |  | -31.14% |
| 31-60 | 13.31% | 7.72% | -5.59% | -9.18% to -2.00% |  | -42.00% |
| 61-180 | 19.96% | 22.60% | 2.64% | -2.20% to 7.48% |  | 13.23% |
| 181-365 | 8.76% | 11.30% | 2.54% | -1.01% to 6.09% |  | 29.00% |
| 366-730 | 5.08% | 9.79% | 4.71% | 1.61% to 7.81% |  | 92.72% |
| 731+ | 6.13% | 16.38% | 10.25% | 6.54% to 13.96% |  | 167.21% |

* Chi square test for difference between 1995 and 2009

Supplementary figure 1: Best fitting join-point model of prevalence of antidepressant prescriptions per 1,000 person years


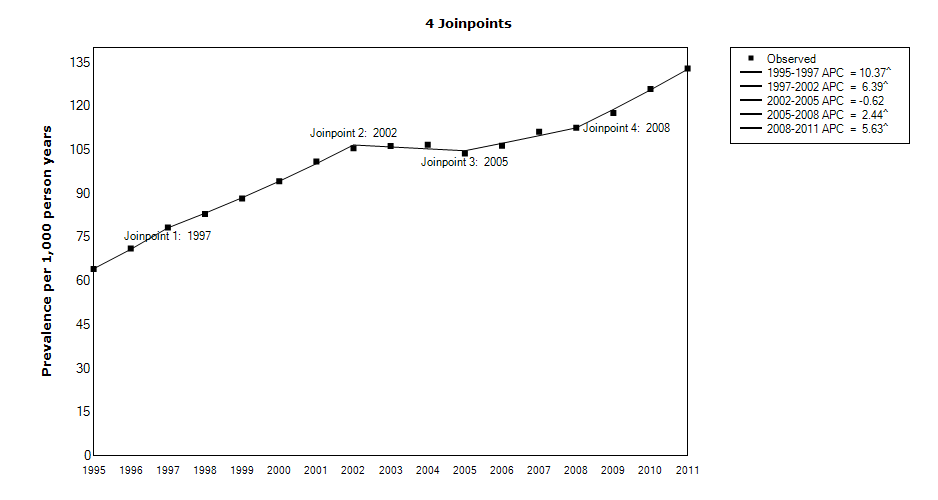


Supplementary figure 2: Best fitting join-point model of incidence of antidepressant prescriptions per 1,000 person years


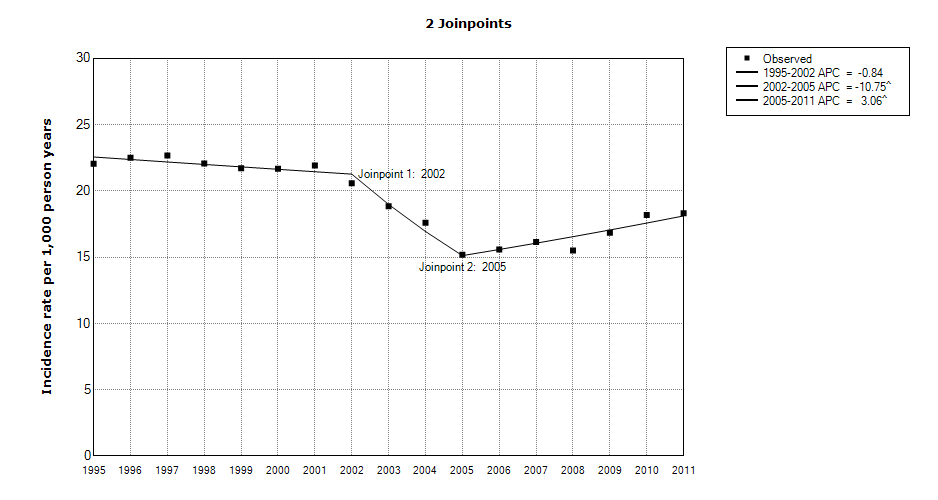


Supplementary figure 3: Incidence of antidepressant prescriptions per 1,000 person years, according to gender

Supplementary figure 4: Incidence of antidepressant prescriptions per 1,000 person years, according to drug class

Supplementary figure 5a: Prevalence of antidepressant prescriptions per 1,000 person years, according to drug class, excluding those prescribed low doses of amitriptyline

Supplementary figure 5b: Incidence of antidepressant prescriptions per 1,000 person years, according to drug class, excluding those prescribed low doses of amitriptyline

Supplementary figure 6a: Prevalence of antidepressant prescriptions per 1,000 person years amongst those with a diagnosis of depression

Supplementary figure 6b: Incidence of antidepressant prescriptions per 1,000 person years, amongst those with a diagnosis of depression
